# Clinical Evaluation of an Abbreviated Contrast-Enhanced Whole-Body MRI for Oncologic Follow-Up Imaging

**DOI:** 10.3390/diagnostics11122368

**Published:** 2021-12-16

**Authors:** Judith Herrmann, Saif Afat, Andreas Brendlin, Maryanna Chaika, Andreas Lingg, Ahmed E. Othman

**Affiliations:** 1 Department of Diagnostic and Interventional Radiology, Eberhard Karls University Tuebingen, Hoppe-Seyler-Strasse 3, 72076 Tuebingen, Germany; judith.herrmann@med.uni-tuebingen.de (J.H.); saif.afat@med.uni-tuebingen.de (S.A.); andreas.brendlin@med.uni-tuebingen.de (A.B.); maryanna.chaika@med.uni-tuebingen.de (M.C.); andreas.lingg@med.uni-tuebingen.de (A.L.); 2 Department of Neuroradiology, University Hospital Mainz, Langenbeckstraße 1, 55101 Mainz, Germany

**Keywords:** magnetic resonance imaging, whole body imaging, cancer staging

## Abstract

Over the last decades, overall survival for most cancer types has increased due to earlier diagnosis and more effective treatments. Simultaneously, whole-body MRI-(WB-MRI) has gained importance as a radiation free staging alternative to computed tomography. The aim of this study was to evaluate the diagnostic confidence and reproducibility of a novel abbreviated 20-min WB-MRI for oncologic follow-up imaging in patients with melanoma. In total, 24 patients with melanoma were retrospectively included in this institutional review board-approved study. All patients underwent three consecutive staging examinations via WB-MRI in a clinical 3 T MR scanner with an abbreviated 20-min protocol. Three radiologists independently evaluated the images in a blinded, random order regarding image quality (overall image quality, organ-based image quality, sharpness, noise, and artifacts) and regarding its diagnostic confidence on a 5-point-Likert-Scale (5 = excellent). Inter-reader agreement and reproducibility were assessed. Overall image quality and diagnostic confidence were rated to be excellent (median 5, interquartile range [IQR] 5–5). The sharpness of anatomic structures, and the extent of noise and artifacts, as well as the assessment of lymph nodes, liver, bone, and the cutaneous system were rated to be excellent (median 5, IQR 4–5). The image quality of the lung was rated to be good (median 4, IQR 4–5). Therefore, our study demonstrated that the novel accelerated 20-min WB-MRI protocol is feasible, providing high image quality and diagnostic confidence with reliable reproducibility for oncologic follow-up imaging.

## 1. Introduction

Over the last decades, overall survival has increased for most cancer types due to earlier diagnosis and more effective treatments, especially due to the rapid progress of new cancer therapies, e.g., immunotherapy [1,2]. A prime example of such an oncologic disease with its recent therapeutic efforts is malignant melanoma. According to the latest research data, patients with metastatic melanoma can have long-term absence of disease after immunotherapy. Therefore, we can suggest a long-term survival rate with no significant difference to normal life expectancy [3].

Diagnostic imaging plays a key role in the surveillance of the patients, especially if there is the status of “no evidence of disease”. Due to its short acquisition time and widespread availability, cancer patients who need repeated examinations for oncologic staging are typically followed with whole-body (WB) computed tomography (CT) or on account of its higher sensitivity for small metastases, positron emission tomography (PET) scan [4,5,6,7]. Accumulated CT scans significantly increase the radiation exposure and the risk of second malignancies due to ionizing radiation. Often, patients are young and have high long-term survival rates so that radiation protection comes to the fore [3].

A potential solution for this is to replace CT scanning through magnetic resonance imaging (MRI). Cancer patients can benefit from the excellent soft-tissue contrast of MR technology without being exposed to ionizing radiation. The disadvantage of this solution is that WB-MRI is time-consuming and is prone to artifacts. Until a few years ago, the general belief was that MRI was not yet ready to face this task due to long scanning times and, therefore, low throughput, as well as high costs of MRI scans.

Through technological advances in hardware and sequence techniques, MRI has enabled the acquisition of WB-MR images in a reduced time without compromising the image quality. Especially in imaging the upper abdomen, new sequence techniques such as parallel imaging and simultaneous multislice (SMS) techniques permit an acceptable degree of temporal and spatial resolution [8,9,10,11,12,13]. Scanner throughput can be increased in order to widen the availability of the diagnostic method to a larger population in a more cost-effective way in daily routine.

The aim of this study is to show that minimized examination time with a fast abbreviated 20-min WB-MRI protocol on a 3 T MRI scanner can provide a reasonable diagnostic image quality and diagnostic accuracy and that this method is providing reliable results.

## 2. Materials and Methods

### 2.1. Study Design

The local ethics committee approved this retrospective study and waived informed patient consent.

Between January and December 2019, patients with clinically indicated WB-MRI as part of the follow-up care were included. Inclusion criteria were defined as age ≥ 18 years, diagnosis of melanoma, with three follow-up examinations via WB-MRI (see Table 1). 

### 2.2. MR System and Imaging Protocol

All examinations were performed on a 3 T scanner (MAGNETOM Vida, Siemens Healthcare, Erlangen, Germany) with patients in a supine position using a 208-channel coil setup (2 × 18-channel body array coils, 74-channel spine coil, 36-channel extremity coil and 64-channel head coil). All patients received body-weight adapted intravenous contrast agent (0.1 mmol/kg Gadobutrol (Gadovist^®^, Bayer Healthcare, Berlin, Germany) at a flow rate of 1.5 mL/s, followed by a saline flush (20 mL). The accelerated 20-min-examination protocol comprised the following sequences: axial T1-weighted (T1w) turbo sin echo (TSE) Dixon of the neck, coronal T2-weighted (T2w) half Fourier single-shot turbo spin-echo (HASTE) of the upper abdomen, simultaneous multislice (SMS) diffusion-weighted imaging (DWI) of the abdomen and pelvis, and axial pre- and post-contrast T1w volumetric interpolated breath-hold examination (VIBE) Dixon from thorax to pelvis (see below, Table 2).

The time needed to execute all the sequences of our 20-min WB-MRI protocol is 10:48 min (Table 2). The real MR examination time for our screening protocol is 20 min including the necessary image reconstruction intervals and shimming times.

### 2.3. Image Analysis

All imaging data were anonymized and randomized, and readers were blinded to the patient history and the radiological report. Three radiologists with ten years, six years and four years of experience in MRI, respectively, independently assessed the abbreviated WB-MRI protocol regarding the following criteria: image quality of each sequence; the organ-based image quality of the following organs—liver, bone, lymph nodes, lung, and cutaneous system; artifacts; and sharpness. Furthermore, overall image quality and diagnostic confidence was evaluated for each examination. Image quality ratings were performed on an ordinal 5-point Likert scale (1, non-diagnostic; 2, poor image quality, non-diagnostic; 3, moderate image quality, diagnostic; 4, good image quality, diagnostic; and 5, excellent image quality, diagnostic). Reading scores were only considered as diagnostic if reaching ≥3. Image analyses were performed on a PACS workstation (GE Healthcare Centricity™ PACS RA1000).

### 2.4. Statistical Analysis

Statistical analyses were performed using SPSS version 26 (IBM Corp, Armonk, NY, USA). Normally distributed values were given as mean ± standard deviation, and non-normally distributed values as the median and interquartile range (IQR). For inter-reader agreement, an intraclass correlation coefficient (ICC) was calculated. Reproducibility was defined as follow-up MRI within the same patient and reproducibility analyses were performed using ICC. ICC-values were interpreted as followed: less than 0.5, poor; 0.5 to 0.75, moderate; 0.75 to 0.9, good; and 0.9 to 1.0, excellent.

## 3. Results

### 3.1. Patients’ Characteristics

A final sample of 24 patients (mean age 54 ± 16, 14 females, 10 males) with three clinically indicated WB-MRI examinations, equalling a total of 72 MRI examinations, was included in this study (see Table 1).

### 3.2. Image Analyses

All three radiologists evaluated the overall image quality in all 72 cases to be excellent (median 5, IQR 5–5), with no rating below 4 points (good image quality, diagnostic). Sharpness of the anatomic structures, as well as the extent of noise of the images, were also rated to be good or excellent in all datasets of all three readers (median 5, IQR 4–5). No relevant artifacts occurred as the item artifacts were rated to be excellent (median 5, IQR 4–5) with just one data set rated with 3 points (moderate image quality, diagnostic) and all others with ratings between good and excellent image quality.

**Figure 1:**
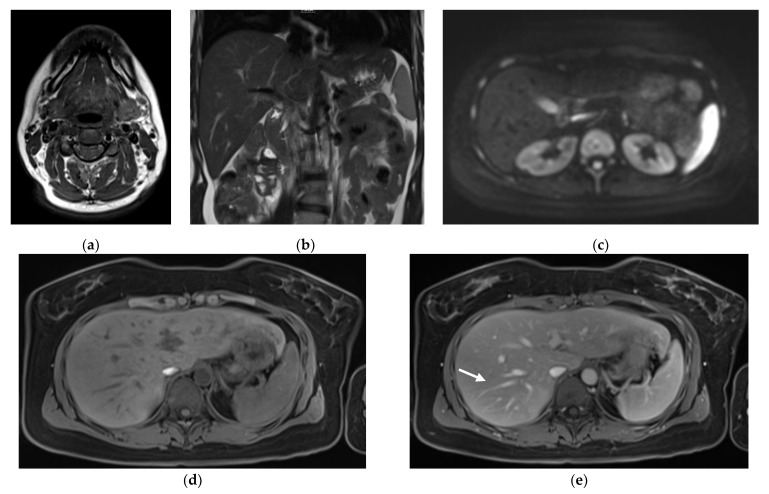
Images illustrating the 20-min-WB-MRI-protocol: example of axial T1-weighted (T1w) TSE Dixon of the neck (**a**), coronal T2-weighted (T2w) HASTE of the upper abdomen (**b**), simultaneous multislice diffusion-weighted imaging of the abdomen and pelvis (**c**), and axial pre- (**d**) and post-contrast T1w VIBE Dixon from thorax to pelvis (**e**) in a 36-year old patient with malignant melanoma and currently no evidence of disease. Note that the sharpness of the anatomic structures, such as the liver vessels (arrow), was rated as excellent. Please note that the images have been cropped, as the skin tissue was part of the acquisition.

In terms of the evaluation of the constituent sequences of the protocol, T2w HASTE of the upper abdomen in coronal orientation and the T1w TSE Dixon in axial orientation of the neck, as well as axial pre- and post-contrast T1w VIBE Dixon were considered by all three radiologists to provide excellent image quality in all datasets (median 5, IQR 4-5). All three radiologists evaluated the axial SMS DWI to display good image quality (median 4, IQR 4–5). Examples of the image quality of the sequences are exhibited in Figure 1. 

**Figure 2:**
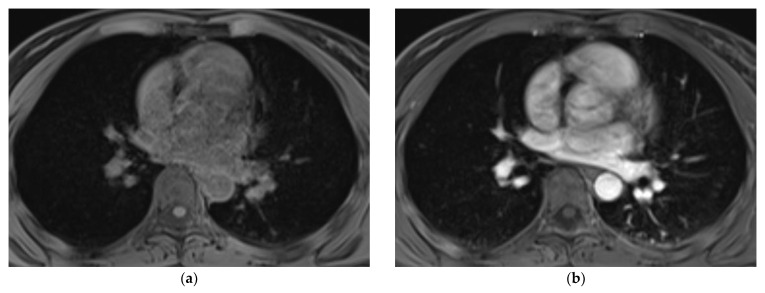
Example of the assessment of the lung in the 20-min-WB-MRI-protocol: example of axial pre- (**a**) and post-contrast T1w VIBE Dixon of the thorax (**b**) in a 25-year old patient with malignant melanoma and currently no evidence of disease. Please note that the images have been cropped, as the skin tissue was part of the acquisition.

**Figure 3:**
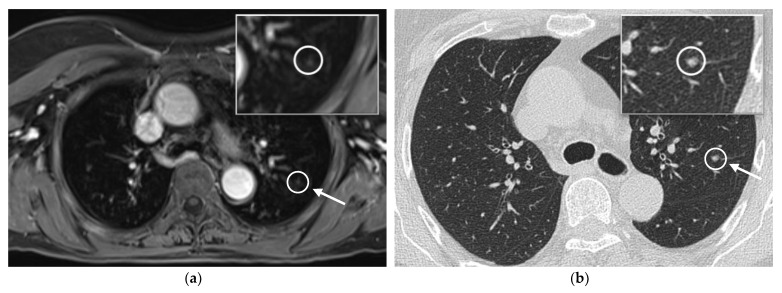
Example of the assessment of the lung in the 20-min-WB-MRI-protocol and the recommended further CT-scan: example of axial post-contrast T1w VIBE Dixon of the thorax (**a**) and CT-scan one week later (**b**) in a 73-year old patient with malignant melanoma and currently no evidence of disease. In the WB-MRI scan, a new pulmonary nodule (arrow) was detected and a further CT scan was recommended. Please note that the images have been cropped, as the skin tissue was part of the acquisition.

With regard to the assessment of the individual organ systems, all three radiologists rated the assessment of lymph nodes, liver, and bone, as well as of the cutaneous system to be excellent (median 5, IQR 4–5), with no rating below 4 points (excellent—good image quality, diagnostic). The image quality of the lung was rated to be good (median 4, IQR 4–5), with ratings between 3 (moderate image quality, diagnostic) and 5 points (excellent image quality, diagnostic). No data set was rated to be non-diagnostic concerning the assessment of the lung. Nonetheless, in 8.3% (6 cases out of 72), a further CT-scan of the lung was recommended (ICC = 0.932). The diagnostic confidence of the WB-MRI data sets was rated to be excellent for all three readers (median 5, IQR 5–5) with no rating below 4 points (excellent—good image quality, diagnostic). Examples of the image quality of the thorax and lung are exhibited in Figure 2 and Figure 3.

Inter-reader agreement ranged between good and excellent with values between 0.833 and 0.924. Reproducibility analysis pointed out that for all items throughout the three evaluated time points, the image quality offered consistent results.

A summary of all descriptive values of the qualitative image analysis is provided in Table 3.

## 4. Discussion

In this study, we investigated an abbreviated 20-min WB-MRI protocol on a 3 T MRI scanner for the staging of patients with malignant melanoma. The results of this study indicate the feasibility of this 20-min WB-MRI protocol as it provides excellent overall image quality and diagnostic confidence. 

We assessed this protocol on patients with malignant melanoma due to their excellent suitability. In early stages, patients with malignant melanoma have long overall survival, are often of young age, and the tumor shows the metastatic spread in almost every organ, whereas image quality and diagnostic accuracy have particularly stringent requirements. 

Recent studies have already demonstrated that the diagnostic performance of WB-MRI is comparable or even superior to other imaging modalities such as CT and PET in numerous cancers making MRI an important tool in oncologic staging [14,15,16,17,18]. Gradually the use of WB-MRI is to an advanced degree in international guidelines [19,20,21].

The current S3 guideline recommends PET-CT for initial staging in patients with melanoma and distant metastases [21]. Cross-sectional imaging is recommended for follow-up of patients with stage IIC melanoma and above [21]. A fixed modality and examination scheme are not stated. However, MRI has been shown to be a sensitive modality for soft tissue metastases and abdominal structures (lymph nodes, liver, fat, and muscle), and MRI has the highest sensitivity for bone metastases [22,23,24]. The prospective study of Muller-Horvat et al. involving 41 metastatic melanoma patients showed that WB-MRI detected some 40% more lesions than WB-CT and, further, treatment strategies were altered due to WB-MRI findings in 24% of the patients [22]. This is consistent with our findings, as the assessment was rated as good to excellent for the organ systems most commonly affected by metastasis in malignant melanoma, such as the cutaneous system, lymph nodes, liver, and bone. Although Pfannenberg et al. found that the overall accuracy of PET-CT was slightly superior to WB-MRI, WB-MRI was more sensitive in detecting liver, bone, and brain metastases, which is in line with our results, with excellent ratings for the assessment of liver and bone [23]. Petralia et al. showed in their study involving 71 MRI scans a very good diagnostic performance in the detection of extracranial metastases in patients with advanced melanoma [25]. The German Dermatological Society recommends WB-MRI for cross-sectional imaging of advanced malignant melanoma (stage III and IV), indicating the equivalence of this method to WB-CT and PET. Only for the evaluation of the lung, CT examination showed better results [24]. As overall and organ-based image quality were rated to be excellent and no examination was rated non-diagnostic, our rapid MRI protocol covers the detection of soft tissue and abdominal metastases at a high-quality level. Although our results showed slightly lower ratings with regard to the assessment of the lung, image quality was still rated to be good and only in 8.3% there was a recommendation of a further CT-scan of the lung, which might be performed with a low-dose protocol to minimize the exposure to radiation [26]. Thus, this WB-MRI protocol seems helpful to establish WB-MRI in the clinical routine of cancer staging, not only in patients with malignant melanoma. One factor that limits WB-MR scans in clinical routine is its long examination. Moreover, MRI centers performing WB-MRI are rare and the procedure is relatively expensive. As we showed in this study, a scanning time of 20 min is adequate to screen patients with malignant melanoma. Nonetheless, latest technological advances such as compressed sensing, deep learning, and automated user interfaces [27,28,29,30,31,32,33,34], have the potential to furthermore accelerate the scanning and improve the efficiency, which is of high interest for the daily clinical routine and needs to be focused on henceforth. The results of our study are encouraging in reducing the scanning time of WB-MRIs.

Although we scanned every patient three times with the same protocol, the major limitation of this study is the relatively small number of included patients. In addition, this study lacks the comparison to a more detailed protocol or other cross-sectional modalities such as CT or PET-CT. Moreover, the retrospective study design is associated with a certain selection bias. Further prospective studies with larger cohorts are needed to evaluate the robustness and diagnostic performance in patients with other cancer entities and pathologies of this 20-min WB-MRI protocol.

## 5. Conclusions

The results of this study indicate the feasibility of this novel accelerated 20-min WB-MRI protocol as it provides excellent overall image quality and diagnostic confidence for the follow-up staging of patients with malignant melanoma.

## Figures and Tables

**Table 1:** Patients’ characteristics.

Variables	
**Age, mean ± SD, y**	54 ± 16
**Sex, male/female**	10/14
**Cancer diagnosis**	Malignant Melanoma
**AJCC-state**	IIB–IV
**IIB,** n	1 (4%)
**IIIB,** n	7 (29%)
**IIIC,** n	13 (54%)
**IV,** n	3 (13%)
**T-state**	
**Tx**, n	3
T1, n	4
T2, n	3
T3, n	9
T4, n	5
**N-state**	
N0	2
N1	12
N2	9
N3	1
**M-state**	
M0	21
M1	3

SD indicates standard deviation; y, year; AJCC, American joint commission on cancer; and n, number.

**Table 2:** Acquisition parameters of the accelerated whole-body MRI protocol.

Parameters	T1 TSE	T2 HASTE	DWI	T1 VIBE	T1 VIBE pc
**Body part**	neck	upper abdomen	abdomen/ pelvis	thorax/ abdomen/ pelvis	thorax/ abdomen/ pelvis
**Orientation**	axial	coronal	axial	axial	axial
**TA, min**	4:04	1:18	4:54	0:36	0:36
**TE/TR, ms**	10/400–750	85/1500	<60/>3000	1.24/3.87	1.24/3.87
**FA, degree**	150	160	90	9	9
**B-value**			b50 b800		
**Spatial resolution**	256 × 320	211 × 384	104 × 134	180 × 320	180 × 320
**Voxel size, mm**	1.09 × 0.88 × 5	1.3 × 1.04 × 5	2.99 × 2.99 × 5	1.75 × 1.31 × 6	1.75 × 1.31 × 6
**FOV, mm**	280 × 280	274 × 400	325 × 420	288 × 420	288 × 420

MRI indicates magnetic resonance imaging; TSE, turbo spin-echo; HASTE, half Fourier single-shot turbo spin-echo; DWI, diffusion-weighted imaging; VIBE, volume-interpolated breath-hold examination; pc, post-contrast; TA, Acquisition Time; TE/TR, echo time/repetition time; FA, flip angle; and FOV, field of view.

**Table 3:** Image Quality, interreader Agreement and reproducibility.

	Reader 1 Median (IQR)	Reader 2 Median (IQR)	Reader 3 Median (IQR)	ICC (Reader)	ICC (Reproducibility)
**Organ-based image quality**					
**IQ_liver_**	5 (4–5)	5 (4–5)	5 (4–5)	0.924	0.915
**IQ_bone_**	5 (4–5)	5 (4–5)	5 (4–5)	0.833	0.912
**IQ_LN_**	5 (4–5)	5 (4–5)	5 (4–5)	0.924	0.915
**IQ_cutanous_**	5 (4–5)	5 (4–5)	5 (4.25–5)	0.912	0.857
**IQ_lung_**	4 (4–5)	4 (4–5)	4 (4–5)	0.899	0.841
**Overall image quality**					
**IQ_overall_**	5 (5–5)	5 (5–5)	5 (5–5)	0.861	0.788
**IQ_sharpness_**	5 (4–5)	5 (4–5)	5 (4–5)	0.834	0.824
**IQ_noise_**	4 (4–5)	5 (4–5)	5 (4–5)	0.865	0.789
**IQ_artifacts_**	5 (4–5)	5 (4–5)	5 (4–5)	0.886	0.813
**IQ_DC_**	5 (5–5)	5 (5–5)	5 (5–5)	0.873	0.857
**Image quality of the sequences**					
**IQ_T1_neck_**	5 (4–5)	5 (4–5)	5 (4–5)	0.892	0.917
**IQ_HASTE_**	5 (4–5)	5 (4–5)	5 (4–5)	0.857	0.855
**IQ_DWI_**	4 (4–5)	4 (4–5)	4 (4–5)	0.894	0.915
**IQ_VIBE_**	5 (4–5)	5 (4–5)	5 (4–5)	0.890	0.890
**IQ_VIBE pc_**	5 (4–5)	5 (4–5)	5 (4–5)	0.919	0.901

IQ indicates image quality; ICC, intraclass correlation coefficient; LN, lymph nodes; DC, diagnostic confidence, DWI, diffusion-weighted imaging; pc, post-contrast; and IQR, interquartile range.

## Data Availability

The data presented in this study are available on request from the corresponding author.
